# Munich cCT Rule for Patients with Recreational Drug and Ethanol Poisoning

**DOI:** 10.3390/jcm13237096

**Published:** 2024-11-24

**Authors:** Tobias Zellner, Felix Wegscheider, Michael Dommasch, Florian Eyer, Rebecca Dieminger, Sabrina Schmoll

**Affiliations:** 1 Division of Clinical Toxicology and Poison Centre Munich, Department of Internal Medicine II, TUM School of Medicine and Health, Technical University of Munich, 81675 Munich, Germany; felix@wegscheider-online.de (F.W.); florian.eyer@tum.de (F.E.); rebecca.dieminger@tum.de (R.D.); sabrina.schmoll@mri.tum.de (S.S.); 2 Central Interdisciplinary Emergency Department, TUM School of Medicine and Health, Technical University of Munich, 81675 Munich, Germany; michael.dommasch@mri.tum.de

**Keywords:** head trauma, cranial computed tomography, drug poisoning, poisoning, Canadian CT head rule, National Emergency X-Radiography Utilization Study Head CT Decision Instrument, New Orleans Criteria, Munich Rule

## Abstract

**Background:** Patients with recreational drug and ethanol poisoning often present with reduced consciousness, coma, or disorientation. It is often unclear if there was recent head trauma. Algorithms to perform cranial computed tomography (cCT) like the Canadian CT Head Rule (CCHR), the National Emergency X-Radiography Utilization Study Head CT Decision Instrument (NEXUS DI), or the New Orleans Criteria (NOC) exist for patients with head trauma. It is unclear whether these algorithms can be applied to this patient collective. **Methods:** This is a retrospective data analysis of patients admitted to our emergency department with drug or ethanol poisoning in 2019. Minors < 16 years were excluded. The primary outcome was fracture/bleeding in cCT, the secondary outcome was neurosurgical intervention. These results were calculated: 1. Sensitivity and negative predictive value (NPV) of the CCHR, NEXUS DI, and NOC. 2. Uni- and multivariate analysis of risk factors for critical findings. 3. The Munich cCT Rule sensitivity and NPV. **Results:** A total of 420 patients were included. cCT was performed in 120 patients. Eight patients had fracture/bleeding in cCT, two required neurosurgical intervention. The number of patients at risk, sensitivity, and NPV for critical cCT findings were as follows: CCHR 57/25%/98.3%, NEXUS DI 239/100%/100%, NOC 420/100%/100%. The sensitivity and NPV for neurosurgical intervention were as follows: CCHR 50%/99.7%, NEXUS DI 100%/100%, NOC 100%/100%. In univariate analysis, these findings correlated significantly with the following critical findings: accident, injury, injury above clavicle, head wound, anisocoria, ethanol in serum > 2 g/L, hypotension, drug ingestion, GCS < 8, focal neurological deficit, age > 60, and cerebellar symptoms. Via chi-square recursive partitioning analysis, we created the Munich cCT Rule which is positive for intoxicated patients if both an accident and an ethanol level > 2 g/L are present. This identified 70 patients at risk. It excluded fracture/bleeding and neurosurgical intervention with a sensitivity and NPV of 100%. **Conclusions:** Fracture/bleeding in cCT in intoxicated patients is rare. Performing unnecessary cCTs should be avoided. The Munich cCT Rule for patients with recreational drug and ethanol poisoning may help rule out critical findings and is superior to the NEXUS DI and NOC. It also has a 100% sensitivity which the CCHR (25%) is lacking.

## 1. Introduction

Recreational drug poisoning is a common problem in emergency departments (EDs). Between 2013 and 2020 alone, 54,314 drug related presentations occurred in the 36 sentinel hospitals of the Euro-DEN network in 24 countries [1]. These patients are usually male (approximately above 75%), in their mid-30s, and have psychiatric co-morbidities [1,2,3,4]. Patients with recreational drug poisoning often present with reduced consciousness, coma, or disorientation. It is often unclear if there was a recent head trauma. Algorithms to perform cranial computed tomography (cCT) like the Canadian CT Head Rule (CCHR) [5], the National Emergency X-Radiography Utilization Study Head CT Decision Instrument (NEXUS DI) [6], or the New Orleans Criteria (NOC) [7] exist for patients with head trauma. These algorithms are designed to identify patients at low risk of significant intracranial injury, thereby reducing unnecessary imaging. However, the applicability of these decision rules to intoxicated patients remains uncertain [8]. Intoxicated patients may present with altered mental status because of the substances ingested, which can mimic or mask symptoms of head injury. This overlap in clinical presentation raises questions about the reliability and validity of these algorithms in this specific patient population. Yang et al. published a recent systematic review and found that some elements used in ED guidelines such as anticoagulant use, headache, and intoxication were not predictive of traumatic intracranial hemorrhage [9].

Schroder et al. showed that even with decompressive craniectomy, mortality remains substantial and favorable outcomes are rare [10]. Therefore, it is paramount not to miss cases with intracranial hemorrhage. Thus, the aim of our study was to

1. Calculate the sensitivity and negative predictive value (NPV) of the CCHR, NEXUS DI and NOC in a cohort of intoxicated patients;
2. Identify risk factors for critical findings in cCT; and
3. Create a score for intoxicated patients (Munich cCT Rule) and calculate its sensitivity and NPV.

## 2. Methods

A retrospective data analysis was conducted on patients admitted to the ED of the TUM University Hospital (formerly Klinikum rechts der Isar) in 2019. The study was conducted in accordance with the Declaration of Helsinki and approved by the Ethics Committee of the Technical University of Munich (protocol code 148/21 S-SR, 12 March 2021).

All patients with drug or ethanol poisoning (with an ICD-10 F1x.0 diagnosis) as a main or secondary diagnosis were included. Validity of the diagnosis was checked by the researchers. We excluded minors under the age of 16 and presentations not related to acute ethanol or drug toxicity (e.g., withdrawal, withdrawal seizures, or hepatic encephalopathy). The primary outcome of interest was the presence of fractures or bleeding detected through cranial computed tomography (cCT) scans. Additionally, the secondary outcome assessed was the necessity for neurosurgical intervention.

Data on patient demographics, clinical features, treatment, and outcome were collected by trained medical staff. Ingested drugs were recorded according to patient’s self-report on admission, bystander reports, emergency medical service (EMS) statements, or clinical interpretation of the drugs by the clinicians managing the patient on admission and classified in the following substance classes: amphetamines/3,4-Methylenedioxymethamphetamine (MDMA), benzodiazepines/Z-substances (Zolpidem, Zopiclone), cannabis, cathinones/phenethylamines, cocaine, gamma-butyrolactone (GBL)/gamma-hydroxybutyric acid (GHB), hallucinogens, Ketamine, Pregabalin/Gabapentin, and opiates/opioids. Patients with multiple visits to our department within the study period were included as multiple cases.

Data analysis was performed on demographic data (i.e., age and sex), clinical characteristics, management, and outcome. Accident (any trauma associated via history with an accident, e.g., falling down a flight of stairs, fall with head trauma, motor vehicle or bicycle accident, pedestrian struck by motor vehicle or visible injury), injury, or head trauma were considered positive if there were visible signs of an accident, injury, or head trauma or patient or bystander history revealed these in direct connection to the admission. Treatment included specific interventions, such as intubation, sedation, and antidote therapy or routine medical interventions (“any treatment”). cCT was performed according to an internal standard operating procedure based on the CCHR and the German guidelines by Zock et al. [11] or at the discretion of the treating surgeon or medical doctor. Neurosurgical intervention was performed after the indication was verified by the responsible attending neurosurgeon. Patients not receiving a cCT were clinically deemed fracture/bleeding negative.

Data were collected in Microsoft Excel Version 2410 (Microsoft, Redmond, WA, USA) and analyzed using IBM SPSS Version 29 (IBM, Armonk, NY, USA). Qualitative variables were summarized using absolute numbers and percentages. Quantitative variables are displayed as the median plus interquartile range (IQR) for non-Gaussian distributed variables and as means plus standard deviation for Gaussian-distributed variables. Statistical differences were tested using the chi-square test for qualitative variables, the Wilcoxon–Mann–Whitney U-test for non-Gaussian distributed variables, or the *t*-test for Gaussian distributed quantitative variables. Sensitivity and negative predictive values are displayed with their 95% confidence interval (CI 95%). Results were considered statistically significant if the *p* value was <0.05. Odds ratios and likelihood ratios were calculated using stepwise multinominal logistic regression analysis as described by Eizadi-Mood et al. [12]. Likelihood ratios were used to perform chi-square recursive partitioning analysis to create the Munich cCT Rule according to the methods described by Stiell et al. [13]. The resulting specificity and sensitivity of the Munich CT Rule were compared to the other rules using McNemar’s test.

## 3. Results

A total of 420 patients were included, and 132 (31.4%) were females. cCT was performed in 120 (28.6%) patients, and 112 (26.7%) had a previous known trauma. Eight patients (1.9%) had fractures (*n* = 2) or bleeding (*n* = 6) in cCT findings, two (0.5%) required neurosurgical intervention (Table 1).

The median age was significantly higher in the no fracture/bleeding group (*p* = 0.007). Using the cut-off of 60 years, patients > 60 years were overrepresented in the fracture/bleeding group (*p* = 0.042). All patients in the fracture/bleeding group had a serum ethanol concentration >2 g/L (*p* = 0.008). Patients with recreational drug ingestion (excluding ethanol) had a significantly lower rate of fracture/bleeding in cCT (*p* = 0.024).

Ethanol was the most common ingested substance, followed by benzodiazepines/Z-substances (Zolpidem, Zopiclone), opiates/opioid, Pregabalin/Gabapentin, and cannabis (Table 2). No substance was significantly associated with fracture/bleeding.

Most patients presented with disorientation and a GCS ≤ 14 on admission. A GCS ≤ 8 at any time and hypertension > 140 mmHg on admission were also common. Agitation/aggression, hallucinations, and anxiety were also prominent features requiring specific interventions like sedation, neuroleptic treatment, or fixation. Four patients presented in or after cardiac arrest and 14 patients had respiratory distress. Only hypotension < 90 mmHg (*p* = 0.016) and GCS ≤ 8 on admission (0.036) were significantly associated with fracture/bleeding in cCT (Table 3).

In univariate analysis, the following head trauma-specific findings correlated significantly with critical findings in cCT: accident (*p* < 0.001), injury (*p* < 0.001), injury above clavicle (*p* < 0.001), head wound (*p* < 0.001), anisocoria (*p* < 0.001), focal neurological deficit (*p* = 0.041), and cerebellar symptoms (*p* = 0.048) (Table 3).

Only 28.8% of patients required any medical treatment. A total of 59 patients were transferred to an intensive care unit (ICU), three required cardiopulmonary resuscitation, and one patient died in the hospital. The main treatment modality was sedation and application of neuroleptics. A total of 16 patients required intubation, and most patients were adequately treated with naloxone or flumazenil.

Most patients left the clinic against medical advice (AMA) or were discharged regularly. Some patients were referred to psychiatry for further treatment. Only intubation was significantly associated with the outcome since two intubated patients were in the fracture/bleeding group and 14 were in the other group (*p* = 0.002) (Table 4).

Calculating the number of patients at risk for critical cCT findings, sensitivity, and NPV for the CCHR, NEXUS DI, and NOC revealed the results displayed in Table 5.

Stepwise multinominal logistic regression analysis with backward entry was performed. The input was fracture/bleeding as dependent variable. The following binary variables, significant in univariate testing, and not overlapping were included: accident, age > 60 years, anisocoria, cerebellar symptoms, ethanol in serum > 2 g/L, focal neurological deficit, drug ingestion, GCS < 8 at admission, and hypotension < 90 mmHg. The analysis gave the following results:

- The variables age >60 years, drug ingestion, and hypotension < 90 mmHg were removed
- The resulting likelihood ratios (LR) of the remaining variables were 38.4 for accident (*p* < 0.001), 26.9 for ethanol in serum > 2 g/L (*p* = 0.012), 23.9 for GCS < 8 at admission (*p* = 0.066), and 23.3 for anisocoria (*p* = 0.099)

**Figure 1:**
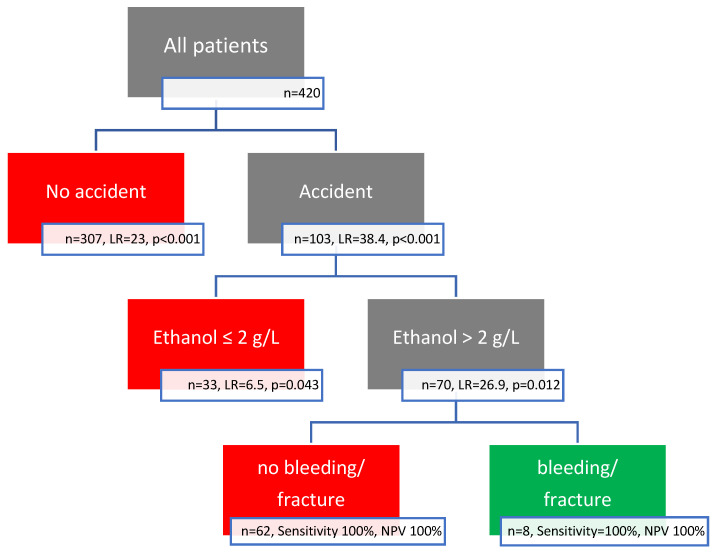
Munich score recursive partitioning analysis result. LR = likelihood ratio.

Via chi-square recursive partitioning analysis, we created the Munich cCT Rule for intoxicated patients. It starts with the variable with the highest likelihood ratio (accident) and continues excluding low risk patients until the target sensitivity and NPV of 100% are not reached anymore. The resulting Munich cCT Rule for intoxicated patients states that patients with recreational drug or ethanol poisoning are at risk for fracture/bleeding if the conditions poisoning, accident, and ethanol > 2 g/L are all met (*n* = 70). All other intoxicated patients were not at risk (*n* = 350). It excluded fracture/bleeding and neurosurgical intervention with a sensitivity of 100% (CI 95% 60–100%) and an NPV of 100% (CI 95% 98.6–100%) for fracture/bleeding and a sensitivity of 100% (CI 95% 19.8–100%) and an NPV of 100% (CI 95% 98.6–100%) for neurosurgical intervention (Table 5, Figure 1).

## 4. Discussion

This study highlights the limitations of established cCT rules like the CCHR, the NEXUS DI, and the NOC when applied to patients with recreational drug or ethanol poisonings who present with reduced consciousness (GCS < 14). These rules were not designed for this specific patient group, leading to significant issues in their application for the patient collective with recreational drug or ethanol poisoning.

In the following section, the respective advantages and limitations of the established cCT rules when applied to intoxicated patients will be discussed.

### 4.1. Canadian CT Head Rule (CCHR) [5]

The CCHR in the patient collective of recreational drug or ethanol poisoning without a known head trauma has a sensitivity issue. This is primarily because the rule includes high-risk factors such as failure to reach a GCS of 15 within two hours, suspected open skull fracture, any sign of basal skull fracture, vomiting for more than two episodes, or age over 65 years. Medium-risk factors include amnesia before impact for more than 30 min and dangerous mechanisms of injury. However, in intoxicated patients, only the dangerous mechanism of injury (pedestrian struck by motor vehicle, occupant ejected from motor vehicle, fall from height >3 feet or five stairs) is a significant risk factor, leading to the rule’s low sensitivity. This was also observed by Ro et al. in the Traumatic Brain Injury Research Network of Korea studying patients with blunt head trauma without poisoning [14]. While the specificity of the CCHR and Munich Rule are comparable, the sensitivity, which is more important in this setting, is superior in the Munich Rule. The NPV, not measured in McNemar’s test, is also superior in the Munich Rule. Many patients present with a GCS < 15 due to their poisoning, leading to an overestimation of the risk.

### 4.2. NEXUS DI [6]

The NEXUS DI in our patient population overestimates the risk of fracture/bleeding. The NEXUS DI rules out critical cCT findings if there is no evidence of skull fracture, scalp hematoma, neurologic deficit, abnormal level of alertness, abnormal behavior, persistent vomiting, coagulopathy, and age over 65 years. Many patients with recreational drug or ethanol poisonings exhibit abnormal levels of alertness or behavior, causing the NEXUS DI to overestimate the risk for critical cCT findings in this cohort. This was also observed by Forouzannia et al. in Iranian patients without intoxication [15]. While the NEXUS DI and the Munich Rule have a similar sensitivity, this difference is reflected in the specificity and also makes the Munich Rule statistically superior when comparing specificity and sensitivity.

### 4.3. New Orleans Criteria (NOC) [7]

Using the NOC would lead to a massive overuse of cCTs since the NOC recommends a cCT if any of the following are present: headache, vomiting, age over 60 years, drug or ethanol poisoning, deficits in short-term memory, physical evidence of trauma above the clavicles, and seizure. This results in all patients with head trauma and intoxication being classified as NOC positive, leading to an overestimation of the need for cCT scans. This high sensitivity resulting in many unnecessary CT scans was also observed by Foks et al. [16]. While the NOC and the Munich Rule have a similar sensitivity, this difference is reflected in the specificity and also makes the Munich Rule statistically superior when comparing specificity and sensitivity.

### 4.4. New Munich cCT Rule for Intoxicated Patients

Based on our retrospective study with 420 patients with recreational drug or ethanol poisoning, we suggest the preliminary Munich cCT Rule for poisoned patients. The Munich cCT Rule for poisoned patients offers a tailored approach for these patients. Based on our data, it only recommends cCTs for approximately 50% of cases compared to the current guidelines used in the emergency department and not designed for these patients (ED). Our rule aims to significantly reduce radiation exposure, particularly for young patients, while maintaining a 100% sensitivity and negative predictive value (NPV). When creating the rule, the prerogative was to not miss critical injuries while avoiding unnecessary radiation exposure. However, due to the rather small patient collective and missing external validation, results should be interpreted with caution.

Our study identified similar risk factors for fractures or bleeding as those included in the CCHR, NEXUS DI, and NOC. However, the application of these rules to poisoned patients is problematic due to the unique presentation of this patient group. Our findings suggest that poisoned patients often present with symptoms that would lead to an overestimation of the need for cCT scans when using traditional rules.

Our study underscores the need for specialized cCT rules for patients with recreational drug or ethanol poisonings who present with reduced consciousness. The Munich cCT Rule for poisoned patients provides a promising alternative ensuring high sensitivity and NPV. This approach may lead to better patient outcomes and more efficient use of medical resources. One challenge is that determining serum ethanol is somewhat time-consuming, and results are often only available after laboratory work is finished. This could lead to a delay in imaging studies and neurosurgical intervention. By only using incident trauma as a criterion for imaging, this would also result in 100% sensitivity and NPV. However, this would result in a 132% increase in cCT scans, exposing more patients to unnecessary radiation and increased expenditure. Since the CCHR has “failure to reach a GCS of 15 within two hours” as a criterion, it would justify waiting until serum ethanol concentration measurements are finished, which normally takes less than two hours.

Many other scores, like the HEAD Rule (“Head trauma”: Head injury or signs of possible head injury, risk assessment using the Canadian CT Head Rule; “Status epilepticus”: not explainable by substance overdose or other syndromes such as myoclonic epilepsy; “Abnormal behavior or neurological signs/symptoms”: not explainable by substance overdose; “Delayed or incomplete neurological recovery or hypoxic brain damage”: e.g., poor recovery without sedation, focal neurological deficits, dilated, fixed pupils.) have been suggested [17]. However, all these scores are based on trauma patients with known head trauma and are not necessarily applicable to unconscious patients without a reliable history with recreational drug or ethanol poisoning.

Serological protein S100B measurement may be helpful as a screening test to identify patients with higher risk of traumatic brain injury for further diagnostic assessment [18]. Serum GFAP and UCH-L1 have also been identified as biomarkers for brain injury [19]. Recently, the neutrophil-albumin ratio has been suggested as a superior prognostic biomarker for traumatic brain injury [20]. Unfortunately, S100B, serum GFAP or UCH-L1, and albumin are not routinely measured in poisoned patients in our ED. These measurements could be included in further studies.

Rotational thromboelastometric (ROTEM) blood-clot analysis results have also been postulated as mortality predictors in traumatic brain injury. In particular, TBI-induced coagulopathy represented by the parameters PLTEM and EASIX in ROTEM analysis was associated with a severe outcome in the study by Băetu et al. [21].

In patients with traumatic brain hemorrhage admitted to the ICU, different parameters are prognostic for the outcome. In the study by Lu et al. age, mechanical ventilation usage, vasoactive agent usage, intracerebral hemorrhage, temperature, respiration rate, white blood cell count, platelet count, red blood cell distribution width, and glucose were predictors of severe outcome [22]. These parameters notably do not overlap with predictive outcomes for intracranial bleeding anymore.

The main limitation of the study is the monocentric, retrospective design. To confirm our findings, a prospective multicenter study design would be desirable. Also, the overall patient number is relatively small and only eight patients were in the primary and two patients were in the secondary outcome groups. This results in some relatively large margins in CI 95%. One patient with drug poisoning and ethanol <2 g/L in the fracture/bleeding group could change the results significantly. Therefore, further research to validate this study is warranted before it can be applied clinically. This rule is not yet validated externally. Without validation, it remains uncertain whether it can be applied universally. Further studies are needed to confirm its general applicability.

## 5. Conclusions

In patients with ethanol or recreational drug poisoning, the occurrence of fractures or bleeding detectable by cCT is relatively rare. Consequently, it is advisable to avoid unnecessary cCT scans, particularly in younger patients, to minimize exposure to radiation. Traditional clinical decision rules such as the CCHR, NEXUS DI, or the NOC have limited applicability in cases involving recreational drug or ethanol poisoning.

To address this gap, the Munich cCT Rule has been developed specifically for patients with recreational drug toxicity and/or ethanol poisoning. This rule is designed to effectively rule out critical findings or the need for neurosurgical interventions, thereby providing a more tailored approach to managing these patients. By applying the Munich cCT Rule, healthcare providers may be guided to make more informed decisions, ensuring that only those patients who truly need further investigation receive it, while avoiding unnecessary procedures for others. However, further research including biomarkers like S100B to validate this study is warranted before it is applied clinically.

## Figures and Tables

**Table 1:** Patient characteristics. EMS = Emergency medical services, ED = emergency department. * *p* < 0.05.

Patient Characteristics	All *n* = 420	No Fracture/Bleeding *n* = 412	Fracture/Bleeding *n* = 8	Significance *p*
Age (years)	39.3 ± 13.8	52.4 ± 15.5	39.1 ± 13.7	0.007 *
Age > 60 years	29 (6.9%)	27 (6.6%)	2 (25%)	0.042 *
Age > 65 years	21 (5%)	20 (4.9%)	1 (12.5%)	0.327
Female	132 (31.4%)	131 (31.8%)	1 (12.5)	0.244
EMS to ED	372 (89%)	364 (88.8%)	8 (100%)	0.315
Ethanol ingestion	331 (78.8%)	323 (78.4%)	8 (100%)	0.139
Ethanol in serum > 2 g/L	226 (53.8%)	218 (52.9%)	8 (100%)	0.008 *
Drug ingestion	218 (51.9%)	217 (52.7%)	1 (12.5%)	0.024 *
>3 substances consumed	58 (13.8%)	58 (14.1%)	0 (0%)	0.253
History of ethanol/drug abuse	317 (75.5%)	311 (75.5%)	6 (75%)	0.975
History of head trauma	19 (4.5%)	18 (4.4%)	1 (12.5%)	0.273

**Table 2:** Ingested substances. MDMA = 3,4-Methyl-enedioxy-methamphetamine, GBL = Gamma-Butyrolactone, GHB = gamma hydroxybutyrate.

Substances Ingested	All *n* = 420	No Fracture/Bleeding *n* = 412	Fracture/Bleeding *n* = 8	Significance *p*
Ethanol	331 (78.8%)	323 (78.4%)	8 (100%)	0.139
Benzodiazepines/Z-Substances	123 (29.3%)	123 (29.9%)	0 (0%)	0.066
Opiates/Opioids	116 (27.6%)	116 (28.2%)	0 (0%)	0.078
Pregabalin/Gabapentin	82 (19.5%)	82 (19.9%)	0 (0%)	0.160
Cannabis	72 (17.1%)	72 (17.5%)	0 (0%)	0.194
Cocaine	42 (10%)	41 (10%)	1 (12.5%)	0.812
Amphetamines/MDMA	32 (7.6%)	32 (7.8%)	0 (0%)	0.412
Hallucinogens	7 (1.7%)	7 (1.7%)	0 (0%)	0.710
Cathinones/Phenethylamines	7 (1.7%)	7 (1.7%)	0 (0%)	0.710
GBL/GHB	6 (1.4%)	6 (1.5%)	0 (0%)	0.731
Ketamine	2 (0.5%)	2 (0.5%)	0 (0%)	0.843

**Table 3:** Symptoms on admission and during hospital stay. GCS = Glasgow Coma Scale, o.a. = on admission, CT = computed tomography. * *p* < 0.05.

	All *n* = 420	No Fracture/Bleeding *n* = 412	Fracture/Bleeding *n* = 8	Significance *p*
**Symptoms**				
Cardiac arrest o.a.	4 (1%)	4 (1%)	0 (0%)	0.779
Respiratory distress o.a.	14 (3.3%)	14 (3.4%)	0 (0%)	0.596
Hypertension > 140 mmHg o.a.	107 (25.5%)	106 (25.7%)	1 (12.5%)	0.395
Hypotension < 90 mmHg o.a.	7 (1.7%)	6 (1.5%)	1 (12.5%)	0.016 *
GCS ≤ 14 o.a.	210 (52.1%)	204 (51.6%)	6 (75%)	0.190
GCS ≤ 8 o.a.	52 (12.9%)	49 (12.4%)	3 (37.5%)	0.036 *
GCS ≤ 8 at any time	76 (18.1%)	73 (17.7%)	3 (37.5%)	0.150
Disorientation o.a.	199 (56.9%)	192 (56.1%)	7 (87.5%)	0.077
Vomiting o.a.	14 (3.3%)	14 (3.4%)	0 (0%)	0.596
Repeated vomiting ≥ 2	8 (1.9%)	8 (1.9%)	0 (0%)	0.691
Hyperthermia > 38.5 °C o.a.	2 (0.5%)	2 (0.5%)	0 (0%)	0.843
Hypothermia < 36 °C o.a.	56 (13.3%)	56 (13.6%)	0 (0%)	0.263
Anxiety o.a.	42 (10%)	42 (10.2%)	0 (0%)	0.341
Agitation/aggression o.a.	84 (20%)	84 (20.4%)	0 (0%)	0.153
Hallucination/Psychosis o.a.	26 (6.2%)	26 (6.3%)	0 (0%)	0.463
Palpitations o.a.	1 (0.2%)	1 (0.2%)	0 (0%)	0.889
Chest pain o.a.	7 (1.7%)	7 (1.7%)	0 (0%)	0.710
**Head trauma predictors**				
Headache o.a.	6 (1.4%)	6 (1.5%)	0 (0%)	0.731
Amnesia o.a.	30 (7.1%)	30 (7.1%)	0 (0%)	0.428
Seizure before CT	20 (4.8%)	19 (4.6%)	1 (12.5%)	0.299
Anisocoria	13 (13.1%)	11 (2.7%)	2 (25%)	<0.001 *
Focal neurological deficit	9 (2.1%)	8 (1.9%)	1 (12.5%)	0.041 *
Cerebellar symptoms	30 (7.1%)	28 (6.8%)	2 (25%)	0.048 *
Visual or auditory impairment	3 (0.7%)	3 (0.7%)	0 (0%)	0.809
Accident	103 (24.5%)	95 (23.1%)	8 (100%)	<0.001 *
Dangerous accident mechanism	7 (1.7%)	6 (1.5%)	1 (12.5%)	0.016 *
Injury	92 (21.9%)	85 (20.6%)	7 (87.5%)	<0.001 *
Injury above clavicle	72 (17.1%)	65 (15.8%)	7 (87.5%)	<0.001 *
Head wound	60 (14.3%)	54 (13.1%)	6 (75%)	<0.001 *
Open head fracture	0 (0%)	0 (0%)	0 (0%)	-
Sign of skull base fracture	0 (0%)	0 (0%)	0 (0%)	-
Liquorrhea	0 (0%)	0 (0%)	0 (0%)	-
Monocular or retroarticular hematoma	12 (2.9%)	12 (2.9%)	0 (0%)	0.624
History of head trauma	19 (4.5%)	18 (4.4%)	1 (12.5%)	0.273
Oral anticoagulation/coagulopathy	14 (3.3%)	13 (3.2%)	1 (12.5%)	0.145

**Table 4:** Treatment and outcome. CPR = cardiopulmonary resuscitation, AMA = against medical advice, Disc. Dc. = disciplinary discharge. * *p* < 0.05.

Treatment and Outcome	All *n* = 420	No Fracture/Bleeding *n* = 412	Fracture/Bleeding *n* = 8	Significance *p*
Treatment required	121 (28.8%)	118 (28.6%)	3 (37.5%)	0.584
Intensive care required	59 (14%)	56 (13.6%)	3 (37.5%)	0.054
CPR required	3 (0.7%)	3 (0.7%)	0 (0%)	0.809
Intubation	16 (3.8%)	14 (3.4%)	2 (25%)	0.002 *
Sedation	79 (18.8%)	77 (18.7%)	2 (25%)	0.651
Neuroleptics	13 (3.1%)	13 (3.2%)	0 (0%)	0.610
Naloxone	23 (5.5%)	23 (5.6%)	0 (0%)	0.492
Flumazenil	15 (3.6%)	15 (3.6%)	0 (0%)	0.583
In-hospital death	1 (0.2%)	1 (0.2%)	0 (0%)	0.889
Discharge Regular	146 (34.8%)	141 (34.2%)	5 (62.5%)	0.789
Psychiatry	28 (6.7%)	28 (6.8%)	0 (0%)	
AMA	224 (53.3%)	221 (53.6%)	3 (37.5%)	
Disc. dc.	11 (2.6%)	11 (2.7%)	0 (0%)	
Runaway	9 (2.1%)	9 (2.2%)	0 (0%)	
unknown	1 (0.2%)	1 (0.2%)	0 (0%)	
Death	1 (0.2%)	1 (0.2%)	0 (0%)	

**Table 5:** Scores—sensitivity and negative predictive value. CI = confidence interval, CCHR = Canadian CT Head Rule, NEXUS DI = National Emergency X-Radiography Utilization Study Head CT Decision Instrument, NOC = New Orleans Criteria.

Score	At Risk *n* = 420 (%)	Specificity	Sensitivity % (CI 95%)	Negative Predictive Value % (CI 95%)	Significance vs. Munich CT Rule
For fracture/bleeding					
CCHR	57 (13.6%)	86.6% (82.8–89.7%)	25% (4–64%)	98.3% (96.2–99.3%)	0.34
NEXUS DI	239 (56.9%)	30.2% (25.4–35.5%)	100% (60–100%)	100% (95.4–100%)	<0.001
NOC	420 (100%)	0.2% (0–1.6%)	100% (60–100%)	100% (5.5–100%)	<0.001
Munich cCT Rule	70 (16.7%)	85.0% (81.0–88.2%)	100% (60–100%)	100% (98.6–100%)	-
For neurosurgical intervention					
CCHR	57 (13.6%)	86.6% (82.8–89.6%)	50% (2.7–97.3%)	99.7% (98.2–100%)	0.34
NEXUS DI	239 (56.9%)	29.7% (24.9–34.9%)	100% (19.8–100%)	100% (95.4–100%)	<0.001
NOC	420 (100%)	0.2% (0–1.5%)	100% (19.8–100%)	100% (5.5–100%)	<0.001
Munich cCT Rule	70 (16.7%)	83.7% (80.0–87.1%)	100% (19.8–100%)	100% (98.6–100%)	-

## Data Availability

Data can be requested from the corresponding author.
